# Consensus-Based Power System State Estimation Algorithm Under Collaborative Attack

**DOI:** 10.3390/s24216886

**Published:** 2024-10-27

**Authors:** Zhijian Cheng, Guanjun Chen, Xiao-Meng Li, Hongru Ren

**Affiliations:** 1 School of Automation, Guangdong-Hong Kong Joint Laboratory for Intelligent Decision and Cooperative Control, and Guangdong Provincial Key Laboratory for Intelligent Decision and Cooperative Control, Guangdong University of Technology, Guangzhou 510006, China; chengzhijian2019@163.com; 2 CSSC Systems Engineering Research Institute, China State Shipbuilding Corporation Limited, Beijing 100094, China; guanjunchen@buaa.edu.cn; 3 School of Mechanical and Electrical Engineering, Guangzhou University, Guangzhou 510006, China; lixiaomeng66@163.com

**Keywords:** power systems, distributed state estimation, collaborative attacks, denial of service attack, false data injection attack

## Abstract

Due to its vulnerability to a variety of cyber attacks, research on cyber security for power systems has become especially crucial. In order to maintain the safe and stable operation of power systems, it is worthwhile to gain insight into the complex characteristics and behaviors of cyber attacks from the attacker’s perspective. The consensus-based distributed state estimation problem is investigated for power systems subject to collaborative attacks. In order to describe such attack behaviors, the denial of service (DoS) attack model for hybrid remote terminal unit (RTU) and phasor measurement unit (PMU) measurements, and the false data injection (FDI) attack model for neighboring estimation information, are constructed. By integrating these two types of attack models, a different consensus-based distributed estimator is designed to accurately estimate the state of the power system under collaborative attacks. Then, through Lyapunov stability analysis theory, a sufficient condition is provided to ensure that the proposed distributed estimator is stable, and a suitable consensus gain matrix is devised. Finally, to confirm the viability and efficacy of the suggested algorithm, a simulation experiment on an IEEE benchmark 14-bus power system is carried out.

## 1. Introduction

Due to the requirements of energy conservation and low carbon, modern power systems have incorporated more and more renewable distributed generation, such as distributed photovoltaic generators and decentralized wind power, which inevitably transforms the power system into a complex large-scale system [1,2,3,4]. In order to monitor and control such systems efficiently and flexibly, the supervisory control and data acquisition system needs to be enhanced by utilizing advanced sensing and network technologies, especially the introduction of phasor measurement units (PMUs) and communication networks [5]. Compared with traditional remote terminal units (RTUs), PMUs have the advantage of being able to obtain the magnitude and phase of node voltage at a higher sampling frequency. In addition, by installing a sufficient number of PMUs in power systems, all system states can be observable and easily derived using linear measurement equations. With economic considerations, however, it is not feasible to switch out all of the traditional RTUs with PMUs anytime soon [6]. Consequently, it is important, yet difficult, to investigate power system issues using mixed RTU and PMU measurements.

Since advanced PMUs and communication networks have been widely used in power systems, the dynamic state estimators have been drawing a fast increasing amount of research interest [7,8,9]. In recent years, a large number of dynamic state estimation methods have been proposed, including Kalman filtering [10], robust filtering [11], adaptive filtering [12], etc. In such large-scale complex systems, the dynamic state estimator based on the centralized scheme is computationally burdened by the larger size of states and measurements, making it unsuitable for such systems. Different from the centralized one, the distributed estimation algorithm is more suitable for large-scale power systems due to its advantages in high computational efficiency, low communication burden, and strong robustness [13]. For example, the distributed algorithm based on the weighted least-square issue was proposed in [14,15,16], which was computationally capable of parallel and distributed processing of power system state estimation problems, thus improving the computational burden faced by centralized estimation. In [17], the authors investigated distributed state estimation issues by utilizing Least Absolute Value, improved the computational performance of large-scale power systems, and achieved real-time monitoring. Note that the above algorithms focus mainly on optimizing methods to control events with accuracy and timeliness by minimizing the energy consumption, ignoring the importance of information interaction and calculation in the distributed collaboration process. To compensate for this deficiency, the consensus-based distributed estimation method was proposed in [18,19,20]. The consensus-based distributed estimation algorithm was first designed to handle the distributed filtering problem in sensor networks, which realized the synchronization of estimation information of adjacent nodes by using multi-agent consensus theory and Kalman filter algorithm, also known as the Kalman consensus filter in numerous studies [21,22]. Due to its high reliability and scalability, many variations of consensus-based distributed estimation algorithms have been proposed to deal with various challenges, such as packet loss [23], event-triggered issues [23], and coupled measurements [24]. For large-scale power systems, therefore, investigating the consensus-based distributed estimation algorithm is an important, yet difficult, task.

The distributed nature of modern large-scale power system and the addition of communication networks make it vulnerable to cyber attacks, of which denial of service (DoS) and false data injection (FDI) attacks are the two main categories under which these attacks fall. FDI attacks, as one of the most common types of attacks in power systems, aim to intercept or maliciously change the signal to be transmitted, causing the estimated state to deviate from the actual state of the target [25,26,27], while DoS attacks aim to block the communication channel by frequently sending useless signals, thereby destroying the state estimation process and even making the system unstable in severe cases [28]. Various research results on FDI and DoS attacks for power systems are reported in recent years. For example, the security issue of dynamic state estimations in power systems was explored in [29] when sensors were compromised by completely stealthy FDI attacks. From the attacker’s perspective, the authors of [30] first designed an FDI attack strategy against power system state estimation, and then proposed an attack detection scheme based on deep reinforcement learning. In addition, for power systems subject to consecutive DoS attacks, a modified state estimation algorithm was proposed in [31] to defend against DoS attacks. For power grids vulnerable to time-constrained DoS attacks, the work in [32] discussed how to design robust load frequency control method, and provided a formal condition for stability and performance analysis. It is noted that most existing results focus on a single type of attack. However, with the development of communication and sensor technology, the practical power system is increasingly subject to multiple heterogeneous cyber attacks. Moreover, the distributed state estimation problem is further complicated by the influence of heterogeneous collaborative attacks because different types of attacks are difficult to handle uniformly.

Inspired by the above discussion on power systems, this paper attempts to address the consensus-based distributed estimation issue for power systems subject to heterogeneous collaborative attacks by utilizing mixed RTU and PMU measurements. The following is a summary of the main contributions of this paper:

1. A distributed estimation algorithm is proposed to deal with the dynamic state estimation problem of power system under FDI and DoS collaborative attacks. The considered collaborative attacks directly act on variables such as voltage, current, and power in the power system, which has stronger practical significance.
2. Different from [33], which only considered FDI attacks, and [34], which only considered DoS attacks, the designed distributed consensus estimator can still maintain high accuracy under the influence of FDI and DoS collaborative attacks. In addition, considering the actual deployment issues in large-scale power systems, this paper devises the suboptimal distributed estimator through an approximate method, which greatly reduces the computational cost.
3. This paper theoretically proves that the proposed distributed consensus estimator is stable, as long as the given conditions are satisfied by jointly designing the consensus gain matrix, collaborative attack parameters, and system model parameters.

The following will introduce the structure of the rest of the paper. Section 2 covers system and attack models, and introduces the research objective of the whole paper. Section 3 constructs the power system model based on hybrid RTU and PMU measurements. Section 4 proposes the distributed consensus estimation method under heterogeneous collaborative attacks. Then, Section 5 conducts the stability analysis for the proposed distributed consensus estimator. Finally, Section 6 verifies the effectiveness of the proposed algorithm through simulation experiments, and Section 7 provides the conclusion.

*Notations*: It is hereby declared that the notations used throughout the paper are completely standard. $R^{N}$ means the *N*-dimensional Euclidean space. $A^{T}$ is the transpose of the vector/matrix *A*, while the inverse matrix of an invertible n×n matrix *A* is indicated by $A^{-1}$. tr(B) denotes the trace of the matrix *B*. E{C} and P{C} are the expectation and probability density function of the random variable *C*, respectively. N(μ,R) is used to define the Gaussian random variable with the mean value μ and covariance matrix *R*. $1_{m}$ represents an m-dimensional column vector, where all elements are 1. $0_{m}$ represents an m-dimensional column vector, where all elements are 0.

## 2. Problem Formulation and Preliminaries

### 2.1. System Description

For the purpose of designing a distributed estimator, this paper consider the following linear discrete-time systems:

(1) $$\begin{array}{cc} X_{k+1}= & \;AX_{k}+Bu+w_{k} \end{array}$$

(2) $$\begin{array}{cc} Y_{i,k}= & \;H_{i,k}X_{k}+v_{i,k},\;i=1,2,\cdots ,M \end{array}$$

where $X_{k}\in R^{2N}$ and $Y_{i,k}\in R^{M}$ represent the state variable of the system and the observed variable by the *i*-th sensor, respectively. $u\in R^{2N}$ is the variable that controls the trend behavior of the state trajectory [35]. *A*, *B*, and $H_{i,k}$ are the system, input, and measurement matrices, respectively. The system and measurement noise, denoted by the random variables $w_{k}$ and $v_{i,k}$, respectively, are assumed to be mutually independent, and satisfy $w_{k}∼N\left(0,Q_{k}\right)$ and $v_{i,k}∼N\left(0,R_{i,k}\right)$.

We assume that the sensor network topology used to observe the above system is modeled as a fixed undirected graph $G=(V_{N},E_{N})$, which consists of the index set of all sensors $V_{N}=\left\{V_{1},V_{2},\cdots ,V_{M}\right\}$ and the set of edges $E_{N}\subseteq V_{N}\times V_{N}$. The set containing all neighbors of node *i* is defined as $N_{i}=\left\{j|\left(i,j\right)\in E_{N}\right\}$.

Objectively speaking, distributed power systems have a huge amount of state and measurement data, which causes existing estimators to face severe computational and communication burdens [36]. In terms of that, this paper introduces the same distributed consensus estimator as in [37],

(3) $$\begin{array}{c} {\hat{X}}_{i,k+1}=\;A{\hat{X}}_{i,k}+Bu+K_{i,k}\left(Y_{i,k}-H_{i,k}{\hat{X}}_{i,k}\right)+L_{i,k}\sum_{j\in N_{i}}\left({\hat{X}}_{j,k}-{\hat{X}}_{i,k}\right) \end{array}$$

where ${\hat{X}}_{i,k}$ represents the estimate of $X_{k}$ for the *i*-th estimator at time instant *k*. The matrices $K_{i,k}$ and $L_{i,k}$ denote the estimator gain and consensus gain to be determined, respectively.

### 2.2. Attack Model

As everyone knows, there are two groups of communication channels involved in distributed filtering across sensor networks. Measurement information is transmitted from the sensor to the estimator through one channel, which connects the sensor and the local estimator. Among the estimators is the other channel, which is used to exchange local estimation information. It is considered that attackers with the ability to alter the transmitted data on the channel may launch random attacks against either of the channels [38]. Therefore, in this paper, we consider that two groups of communication channels are subject to collaborative attacks by FDI and DoS.

1. DoS attack: Assume that the innovation $\Delta_{i,k}$ transmitted from the sensor to the estimator is subject to the DoS attack, i.e.,
  (4) $$\begin{array}{cc} \Delta_{i,k}= & Y_{i,k}-H_{i,k}{\hat{X}}_{i,k}\\ \Delta_{i,k}^{a}= & \alpha_{i,k}\Delta_{i,k} \end{array}$$
  where $\Delta_{i,k}^{a}$ denotes the innovation under the DoS attack. The occurrence of DoS attacks is described by the random variable $\alpha_{i,k}$, which satisfies the Bernoulli distribution. Stated differently, $\alpha_{i,k}=0$ indicates that the channel for transmitting innovation is under DoS attack; otherwise, $\alpha_{i,k}=1$. In addition, the probability density function of $\alpha_{i,k}$ is defined as follows:
  $$\begin{array}{cc} P\{\alpha_{i,k}=0\}= & 1-\alpha_{i}\\ P\{\alpha_{i,k}=1\}= & \alpha_{i} \end{array}$$
  Here, $\alpha_{i}$ represents the probability that the transmitted innovation is not attacked, are assumed to be known to estimator designers.
2. FDI attack: Assume that the estimation information transmitted between nodes is subject to the FDI attack, i.e.,
  (5) $$\begin{array}{c} {\hat{X}}_{j,k}^{a}={\hat{X}}_{j,k}+q_{ij,k}\beta_{ij,k} \end{array}$$
  where ${\hat{X}}_{j,k}^{a}$ represents the estimation information of neighbor nodes transmitted when under FDI attack. $q_{ij,k}$ is defined as the decision variable of the FDI attacker. That is, if $q_{ij,k}=1$, the attacker will invade the communication channel between nodes *i* and *j* at time instant *k*, and the estimation information transmitted between nodes *i* and *j* will be injected with a false signal; otherwise, $q_{ij,k}=0$. The $b_{ij,k}$ that represents the injected false signal is a random variable that satisfies the Gaussian distribution with $b_{ij,k}∼N\left(0,B_{ij,k}\right)$. Note that, here, $b_{ij,k}$ is assumed to be uncorrelated with system and measurement noise.

### 2.3. Problem Statement

In this section, taking into account the case where communication channels from the sensor to estimator and between estimators are subject to the DoS and FDI attacks, respectively, the distributed consensus estimation algorithm in (3) has been updated as follows:

(6) $$\begin{array}{cc} {\hat{X}}_{i,k+1}= & \;A{\hat{X}}_{i,k}+Bu+K_{i,k}\alpha_{i,k}\Delta_{i,k}+L_{i,k}\sum_{j\in N_{i}}\left({\hat{X}}_{j,k}^{a}-{\hat{X}}_{i,k}\right) \end{array}$$

As can be seen from the discussion above, this paper aims to find a feasible distributed consensus estimation algorithm to accurately estimate the state of the system under FDI and DoS collaborative attacks.

## 3. Power System Modeling

This paper assumes that the power network functions within quasi-steady states. These steady-state dynamics are generally distinct from the transient ones produced by electromechanical power systems [35]. The power system state considered in this paper is the bus voltage, which is defined as the vector consisting of the real and imaginary parts of all bus voltages, i.e.,

$$\begin{array}{cc} X_{k}= & {\left[X_{k}^{r},X_{k}^{i}\right]}^{T}\in R^{2N}\\ X_{k}^{r}= & \left[X_{k}^{r,1},X_{k}^{r,2},\cdots ,X_{k}^{r,N}\right]\\ X_{k}^{i}= & \left[X_{k}^{i,1},X_{k}^{i,2},\cdots ,X_{k}^{i,N}\right] \end{array}$$

where $X_{k}^{r}$ and $X_{k}^{i}$ denote the vectors of the real and imaginary voltage, respectively.

Additionally, the power system measurement model constructed in this paper mainly consists of two aspects. One is the measurement $Y_{k}^{\mathrm{rtu}}\in R^{m_{1}}$ collected by RTUs, which is defined as

$$\begin{array}{c} Y_{k}^{\mathrm{rtu}}={\left[V_{k}^{T},P_{k}^{T},Q_{k}^{T},P_{k}^{fT},Q_{k}^{fT}\right]}^{T} \end{array}$$

Further, it has

(7) $$\begin{array}{cc} V_{s,k}= & \sqrt{{\left(X_{k}^{r,s}\right)}^{2}+{\left(X_{k}^{i,s}\right)}^{2}}\\ Q_{s,k}= & X_{k}^{i,s}\sum_{j\in B_{s}}\left(R^{sj}X_{k}^{r,j}-I^{sj}X_{k}^{i,j}\right)-X_{k}^{r,s}\sum_{j\in B_{s}}\left(R^{sj}X_{k}^{i,j}+I^{sj}X_{k}^{r,j}\right)\\ P_{s,k}= & X_{k}^{r,s}\sum_{j\in B_{s}}\left(R^{sj}X_{k}^{r,j}-I^{sj}X_{k}^{i,j}\right)+X_{k}^{i,s}\sum_{j\in B_{s}}\left(R^{sj}X_{k}^{i,j}+I^{sj}X_{k}^{r,j}\right)\\ P_{st,k}^{f}= & \left({\left(X_{k}^{r,s}\right)}^{2}+{\left(X_{k}^{i,s}\right)}^{2}\right)\left(g^{st0}+g^{st}\right)-X_{k}^{r,s}X_{k}^{r,t}g^{st}\\ \; & -X_{k}^{i,s}X_{k}^{i,t}g^{st}-X_{k}^{i,s}X_{k}^{r,t}d^{st}+X_{k}^{r,s}X_{k}^{i,t}d^{st}\\ Q_{st,k}^{f}= & X_{k}^{r,s}X_{k}^{i,t}g^{st}-\left({\left(X_{k}^{r,s}\right)}^{2}+{\left(X_{k}^{i,s}\right)}^{2}\right)\left(d^{st0}+d^{st}\right)\\ \; & -X_{k}^{i,s}X_{k}^{r,t}g^{st}+X_{k}^{r,s}X_{k}^{r,t}d^{st}+X_{k}^{i,s}X_{k}^{i,t}d^{st} \end{array}$$

where $V_{k}={\left[V_{1,k},V_{2,k},\cdots ,V_{N_{v},k}\right]}^{T}$, $P_{k}={\left[P_{1,k},P_{2,k},\cdots ,P_{N_{p},k}\right]}^{T}$, $Q_{k}={\left[Q_{1,k},Q_{2,k},\cdots ,Q_{N_{p},k}\right]}^{T}$, $P_{k}^{f}={\left[P_{1,k}^{f},P_{2,k}^{f},\cdots ,P_{N_{f},k}^{f}\right]}^{T}$, and $Q_{k}^{f}={\left[Q_{1,k}^{f},Q_{2,k}^{f},\cdots ,Q_{N_{f},k}^{f}\right]}^{T}$ denote the voltage magnitude measurement vector, the real and reactive bus power injections measurement vector, and the real and reactive transmission line power flows measurement vector, respectively. Additionally, $R^{sj}+jI^{sj}$, $g^{st}+jd^{st}$, and $g^{st0}+jd^{st0}$ are regarded as the bus admittance connected to bus *s*, the series admittance and the half shunt admittance connecting bus *s* and bus *t*, respectively.

Therefore, derived from (7), the RTU measurement equation is summarized as follows:

(8) $$\begin{array}{c} Y_{k}^{\mathrm{rtu}}=f\left(X_{k}\right) \end{array}$$

The other is the measurement $Y_{k}^{\mathrm{pmu}}\in R^{m_{2}}$ collected by PMUs, which is defined as

$$\begin{array}{c} Y_{k}^{\mathrm{pmu}}=\left[Y_{1,k}^{\mathrm{pmu}},Y_{2,k}^{\mathrm{pmu}},\cdots ,Y_{p,k}^{\mathrm{pmu}}\right] \end{array}$$

Further, it has

$$Y_{l,k}^{\mathrm{pmu}}={\left[Y_{r,j,k}^{\mathrm{pmu}},Y_{i,j,k}^{\mathrm{pmu}},Y_{r,t_{1}^{j},k}^{\mathrm{pmu}},Y_{i,t_{1}^{j},k}^{\mathrm{pmu}},\cdots ,Y_{r,t_{N_{l}}^{j},k}^{\mathrm{pmu}},Y_{i,t_{N_{l}}^{j},k}^{\mathrm{pmu}}\right]}^{T}$$

where

$$\begin{array}{cc} Y_{r,j,k}^{\mathrm{pmu}}= & X_{k}^{r,j},\;Y_{i,j,k}^{\mathrm{pmu}}=X_{k}^{i,j}\\ Y_{r,jt,k}^{\mathrm{pmu}}= & \left(X_{k}^{r,j}-X_{k}^{r,t}\right)g^{jt}-\left(X_{k}^{i,j}-X_{k}^{i,t}\right)d^{jt}+X_{k}^{r,j}g^{jt0}-X_{k}^{i,j}d^{jt0}\\ Y_{i,jt,k}^{\mathrm{pmu}}= & \left(X_{k}^{i,j}-X_{k}^{i,t}\right)g^{jt}+\left(X_{k}^{r,j}-X_{k}^{r,t}\right)d^{jt}+X_{k}^{i,j}g^{jt0}+X_{k}^{r,j}d^{jt0} \end{array}$$

Immediately afterwards, the PMU measurement equation is derived as follows:

(9) $$\begin{array}{c} Y_{k}^{\mathrm{pmu}}=H_{k}^{\mathrm{pmu}}X_{k} \end{array}$$

In this paper, combining (8) and (9), the following hybrid PMU and RTU measurement model is considered:

(10) $$\begin{array}{c} Y_{k}=F\left(X_{k}\right)+V_{k} \end{array}$$

where $Y_{k}={\left[{\left(Y_{k}^{\mathrm{rtu}}\right)}^{T},{\left(Y_{k}^{\mathrm{pmu}}\right)}^{T}\right]}^{T}\in R^{m}$. $V_{k}$ is the introduced noise variable. Furthermore, the linear power system measurement equation can be generated as follows by applying the same linearization technique as in [36,39],

(11) $$\begin{array}{c} Y_{k}=H_{k}X_{k}+v_{k} \end{array}$$

where $H_{k}=\frac{\partial F}{\partial X}{|}_{X_{0,k}}$, and $X_{0,k}$ stands for the current operating point at time instant *k*. $v_{k}=\left(\frac{\partial F}{\partial V}{|}_{V_{0,k}}\right)V_{k}$.

In power systems, the development of the local subsystem model is required to achieve distributed state estimation. Following [36,40], this section makes the same assumption, which is as follows:

$$\begin{array}{c} Y_{k}={\left[Y_{1,k}^{T},\cdots ,Y_{M,k}^{T}\right]}^{T},\;H_{k}={\left[H_{1,k}^{T},\cdots ,H_{M,k}^{T}\right]}^{T},\;v_{k}={\left[v_{1,k}^{T},\cdots ,v_{M,k}^{T}\right]}^{T} \end{array}$$

and it has

(12) $$\begin{array}{c} Y_{i,k}=H_{i,k}X_{k}+v_{i,k},\;i=1,2,\cdots ,M \end{array}$$

## 4. Distributed Estimation Algorithm

In this section, first let $e_{i,k}$ and $P_{ij,k}$ represent the estimation error of estimator *i* and cross estimation error covariance matrix between estimators *i* and *j*, which are defined as

$$\begin{array}{c} e_{i,k}=\;X_{k}-{\hat{X}}_{i,k},\;P_{ij,k}=E\left\{e_{i,k}e_{j,k}^{T}\right\} \end{array}$$

Considering collaborative attacks, there are

$$\begin{array}{c} e_{i,k}^{a}=\;X_{k}-{\hat{X}}_{i,k}^{a},\;P_{ij,k}^{a}=E\left\{e_{i,k}^{a}{\left(e_{j,k}^{a}\right)}^{T}\right\} \end{array}$$

Following that, in order to obtain the optimal state estimate, it will propose a theorem to derive the proper filter gain matrix and error covariance matrix.

**Theorem** **1.** *For the power system, (1) and (12), with N buses and a fixed undirected communication graph G, considering the collaborative impact of FDI attacks in (5) and DoS attacks in (4), the optimal filter gain to minimize the estimation error covariance is derived by*

$$\begin{array}{cc} K_{i,k}= & \;\left[AP_{i,k}+L_{i,k}\sum_{r\in N_{i}}\left({\hat{P}}_{ri,k}^{a}-P_{i,k}\right)\right]H_{i,k}^{T}\Xi_{i,k}^{-1} \end{array}$$

*where $\Xi_{i,k}=\alpha_{i}H_{i,k}P_{i,k}H_{i,k}^{T}+\alpha_{i}R_{i,k}$.*

**Proof** **of** **Theorem** **1.** Combining Equations (1) and (6) and the above definitions, we can obtain

(13) $$\begin{array}{cc} e_{i,k+1}= & \;\left(A-\alpha_{i,k}K_{i,k}H_{i,k}\right)e_{i,k}+w_{k}-\alpha_{i,k}K_{i,k}v_{i,k}+L_{i,k}\sum_{j\in N_{i}}\left(e_{j,k}^{a}-e_{i,k}\right) \end{array}$$

where

(14) $$\begin{array}{c} e_{j,k}^{a}=X_{k}-{\hat{X}}_{j,k}^{a}=e_{j,k}-q_{ij,k}\beta_{ij,k} \end{array}$$

Next, one can obtain the following estimation error covariance iteration equation:

(15) $$\begin{array}{cc} P_{ij,k+1}= & \;E\{e_{i,k+1}e_{j,k+1}^{T}\}\\ = & \;\left(A-\alpha_{i}K_{i,k}H_{i,k}\right)P_{ij,k}{\left(A-\alpha_{j}K_{j,k}H_{j,k}\right)}^{T}+L_{i,k}\\ \; & \;\times \sum_{r\in N_{i}}\sum_{s\in N_{j}}\left(P_{rs,k}^{a}-{\hat{P}}_{rj,k}^{a}-{\overset{ˇ}{P}}_{is,k}^{a}+P_{ij,k}\right)L_{j,k}^{T}\\ \; & \;+\left(A-\lambda_{i}K_{i,k}H_{i,k}\right)\sum_{s\in N_{j}}\left({\overset{ˇ}{P}}_{is,k}^{a}-P_{ij,k}\right)L_{i,k}^{T}\\ \; & \;+L_{i,k}\sum_{r\in N_{i}}\left({\hat{P}}_{rj,k}^{a}-P_{ij,k}\right){\left(A-\lambda_{j}K_{j,k}H_{j,k}\right)}^{T}\\ \; & \;+K_{i,k}\alpha_{i}\alpha_{j}E\left\{v_{i,k}v_{j,k}^{T}\right\}K_{j,k}^{T}+Q_{k} \end{array}$$

By setting i=j, it can be transformed into

(16) $$\begin{array}{cc} P_{i,k+1}= & \;\left(A-\alpha_{i}K_{i,k}H_{i,k}\right)P_{i,k}{\left(A-\alpha_{i}K_{i,k}H_{i,k}\right)}^{T}\\ \; & +L_{i,k}\sum_{\begin{array}{c} r,s\in N_{i}\\ r\neq s \end{array}}\left(P_{rs,k}^{a}-{\overset{ˇ}{P}}_{ri,k}^{a}-{\hat{P}}_{is,k}^{a}+P_{i,k}\right)L_{i,k}^{T}\\ \; & +\left(A-\alpha_{i}K_{i,k}H_{i,k}\right)\sum_{s\in N_{i}}\left({\hat{P}}_{is,k}-P_{i,k}\right)L_{i,k}^{T}\\ \; & +L_{i,k}\sum_{r\in N_{i}}\left({\overset{ˇ}{P}}_{ri,k}-P_{i,k}\right){\left(A-\alpha_{i}K_{i,k}H_{i,k}\right)}^{T}\\ \; & +L_{i,k}\sum_{r\in N_{i}}\left(P_{rs,k}^{a}-{\overset{ˇ}{P}}_{ri,k}^{a}-{\hat{P}}_{is,k}^{a}+P_{i,k}\right)L_{i,k}^{T}\\ \; & +\alpha_{i}^{2}K_{i,k}R_{i,k}K_{i,k}^{T}+Q_{k} \end{array}$$

where

$$\begin{array}{cc} {\overset{ˇ}{P}}_{ri,k}^{a}= & E\left\{e_{r,k}^{a}e_{i,k}^{T}\right\}=P_{ri,k}\\ {\hat{P}}_{is,k}= & E\left\{e_{i,k}{\left(e_{s,k}^{a}\right)}^{T}\right\}=P_{is,k}\\ P_{rs,k}^{a}= & E\left\{e_{r,k}^{a}{\left(e_{s,k}^{a}\right)}^{T}\right\}=P_{rs,k}+q_{ir,k}^{2}B_{ir,k} \end{array}$$

It should be noted that the expression $\sum_{i=1}^{M}E\{∥X_{k}-{\hat{X}}_{i,k}{∥}^{2}\}$ represents the overall estimation error for all nodes. Thus, the following optimization problem needs to be solved to obtain the optimal filter gain matrix:

$$\begin{array}{c} \min_{K_{i,k}}\mathrm{tr}\left(P_{i,k+1}\right) \end{array}$$

Then, applying matrix calculus operation theory yields, as a result,

(17) $$\begin{array}{cc} \frac{\partial \mathrm{tr}(P_{i,k+1})}{\partial K_{i,k}}= & \;2\left(A-\alpha_{i}K_{i,k}H_{i,k}\right)P_{i,k}{\left(-\alpha_{i}H_{i,k}\right)}^{T}\\ \; & +L_{i,k}\sum_{s\in N_{i}}{\left({\hat{P}}_{is,k}^{a}-P_{i,k}\right)}^{T}{\left(-\alpha_{i}H_{i,k}\right)}^{T}\\ \; & +L_{i,k}\sum_{r\in N_{i}}\left({\overset{ˇ}{P}}_{ri,k}^{a}-P_{i,k}\right){\left(-\alpha_{i}H_{i,k}\right)}^{T}\\ \; & +2\alpha_{i}^{2}K_{i,k}R_{i,k} \end{array}$$

where ${\left({\hat{P}}_{is,k}^{a}\right)}^{T}={\overset{ˇ}{P}}_{ri,k}^{a}$.

With Equation (17) rearranged and $\frac{\partial \mathrm{tr}(P_{i,k+1})}{\partial K_{i,k}}=0$, we can naturally obtain

(18) $$\begin{array}{cc} K_{i,k}= & \;\left[AP_{i,k}+L_{i,k}\sum_{r\in N_{i}}\left({\hat{P}}_{ri,k}^{a}-P_{i,k}\right)\right]H_{i,k}^{T}\Xi_{i,k}^{-1} \end{array}$$

where $\Xi_{i,k}=\alpha_{i}H_{i,k}P_{i,k}H_{i,k}^{T}+\alpha_{i}R_{i,k}$. This finishes the proof of Theorem 1. □

From Equation (18), in order to compute the optimal filter gain matrix $K_{i,k}$, obtaining cross-covariance matrices ${\hat{P}}_{ri,k}^{a}$ beforehand are required, which are generally computationally costly. To compensate for this deficiency, a suboptimal filter gain with $L_{i,k}=0$ is devised, i.e., ignoring the impact of cross-covariance matrices in the filter gain design, to improve the computational burden [41]. In light of this, the distributed consensus estimation algorithm that follows is provided:

(19) $$\begin{array}{cc} {\hat{X}}_{i,k+1}= & \;A{\hat{X}}_{i,k}+Bu+K_{i,k}\alpha_{i,k}\left(Y_{i,k}-H_{i,k}{\hat{X}}_{i,k}\right)+L_{i,k}\sum_{j\in N_{i}}\left({\hat{X}}_{j,k}^{a}-{\hat{X}}_{i,k}\right)\\ {\hat{X}}_{j,k}^{a}= & \;{\hat{X}}_{j,k}+q_{ij,k}\beta_{ij,k}\\ K_{i,k}= & \;\alpha_{i}^{-1}AP_{i,k}H_{i,k}^{T}{\left(H_{i,k}P_{i,k}H_{i,k}^{T}+R_{i,k}\right)}^{-1}\\ P_{i,k+1}= & \;\left(A-\alpha_{i}K_{i,k}H_{i,k}\right)P_{i,k}{\left(A-\alpha_{i}K_{i,k}H_{i,k}\right)}^{T}\\ \; & \;+\alpha_{i}^{2}K_{i,k}R_{i,k}K_{i,k}^{T}+Q_{k} \end{array}$$

**Remark** **1.** *Note that while the calculation complexity can be reduced by the approximations employed in (19), the stability of the proposed distributed estimator remains unaffected. As indicated by simulation results, the introduced error is reasonable. Furthermore, these approximations also help to simplify the structure of the suboptimal method, such that it is consistent with traditional Kalman filter.*

## 5. Stability Analysis

For the proposed suboptimal distributed consensus estimation algorithm in (19), stability analysis is a difficult yet necessary field of research. In order to better carry out this study, the following assumptions and lemmas are proposed.

**Assumption** **A1** ([42])**.** *The system matrix A is non-singular.*

**Assumption** **A2** ([43])**.** *The following inequalities hold for some positive real numbers:*

$$\begin{array}{cc} \underline{a}\leq & ∥A∥\leq \bar{a},\;{\underline{h}}_{i}\leq ∥H_{i,k}∥\leq {\bar{h}}_{i}\\ \underline{q}I\leq & Q_{k}\leq \bar{q}I,\;{\underline{r}}_{i}I\leq R_{i,k}\leq {\bar{r}}_{i}I\\ \; & {\underline{p}}_{i}I\leq P_{i,k}\leq {\bar{p}}_{i}I \end{array}$$

**Lemma** **1** ([44])**.** *There is a random process $V_{k}\left(\zeta_{k}\right)$, as well as real numbers $\bar{\nu },\underline{\nu },\upsilon >0$ and 0<κ≤1, that satisfy the following inequalities:*

(20) $$\begin{array}{cc} \underline{\nu }{∥\zeta_{k}∥}^{2}\leq V_{k}\left(\zeta_{k}\right)\leq & \bar{\nu }{∥\zeta_{k}∥}^{2} \end{array}$$

(21) $$\begin{array}{cc} E\left\{V_{k+1}\left(\zeta_{k+1}\right)|\zeta_{k}\right\}-V_{k}\left(\zeta_{k}\right)\leq & \upsilon -\kappa V_{k}\left(\zeta_{k}\right) \end{array}$$

*and the random process $V_{k}\left(\zeta_{k}\right)$ is exponentially bounded in mean square, and is bounded with probability one.*

Next, the following will present the main results of the stability analysis for the distributed consensus estimator.

**Theorem** **2.** *For the power system, (1) and (12), with N buses and a fixed undirected communication graph G, considering the collaborative impact of FDI attacks in (5) and DoS attacks in (4), as well as the suboptimal distributed consensus estimation algorithm proposed in (19), then under Assumptions 1 and 2, and setting the following condition:*

$$\begin{array}{c} \sum_{j\in N_{i}}{\left({\hat{X}}_{j,k}^{a}-{\hat{X}}_{i,k}\right)}^{T}\left({\hat{X}}_{j,k}^{a}-{\hat{X}}_{i,k}\right)\leq \sigma_{i} \end{array}$$

*where $\sigma_{i}>0,i=1,2,\cdots ,M$, the estimation error $e_{i,k}$ is exponentially bounded in the mean square, and is bounded with the probability one.*

**Proof** **of** **Theorem** **2.** In order to make use of Lemma 1, first construct the following augmented estimation error and estimation error covariance for all estimators:

(22) $$\begin{array}{cc} e_{k}= & \left[e_{1,k}^{T},e_{2,k}^{T},\cdots ,e_{M,k}^{T}\right] \end{array}$$

(23) $$\begin{array}{cc} P_{k}= & \mathrm{diag}\{P_{1,k},P_{2,k},\cdots ,P_{M,k}\} \end{array}$$

Combining Equations (22) and (23), design the following Lyapunov function:

(24) $$\begin{array}{c} V_{k}\left(e_{k}\right)=e_{k}^{T}P_{k}^{-1}e_{k}=\sum_{i=1}^{M}e_{i,k}^{T}P_{i,k}^{-1}e_{i,k} \end{array}$$

With the help of Assumption 2, it can be easily verified that the designed Lyapunov function satisfies the condition defined in inequality (20) in Lemma 1, i.e.,

$$\begin{array}{c} \frac{1}{\bar{p}}∥e_{k}{∥}^{2}\leq V_{k}\left(e_{k}\right)\leq \frac{1}{\underline{p}}{∥e_{k}∥}^{2} \end{array}$$

where $\underline{\nu }=\frac{1}{\bar{p}}$ and $\bar{\nu }=\frac{1}{\underline{p}}$ with $\bar{p}=\max\left\{{\bar{p}}_{1},{\bar{p}}_{2},\cdots ,{\bar{p}}_{n}\right\}$ and $\underline{p}=\min\left\{{\underline{p}}_{1},{\underline{p}}_{2},\cdots ,{\underline{p}}_{n}\right\}$.

The next step is to confirm that if the Lyapunov function meets the second condition stated in Inequality (21) in Lemma 1. Towards this end, Equation (24) needs to be further expanded. Substituting Equation (13) into Equation (24) yields

(25) $$\begin{array}{cc} E\{V_{k+1}\left(e_{k+1}\right)\}= & \;\sum_{i=1}^{M}E\left\{e_{i,k}^{T}{\left(A-\alpha_{i}K_{i,k}H_{i,k}\right)}^{T}P_{i,k+1}^{-1}\left(A-\alpha_{i}K_{i,k}H_{i,k}\right)e_{i,k}\right\}\\ \; & +\sum_{i=1}^{M}E\left\{\sum_{j\in N_{i}}{\left(e_{j,k}^{a}-e_{i,k}\right)}^{T}L_{i,k}^{T}P_{i,k+1}^{-1}L_{i,k}\sum_{j\in N_{i}}\left(e_{j,k}^{a}-e_{i,k}\right)\right\}\\ \; & +\sum_{i=1}^{M}E\{\sum_{j\in N_{i}}{\left(e_{j,k}^{a}-e_{i,k}\right)}^{T}L_{i,k}^{T}P_{i,k+1}^{-1}\left(A-\alpha_{i}K_{i,k}H_{i,k}\right)e_{i,k}\\ \; & +\sum_{i=1}^{M}E\left\{e_{i,k}^{T}{\left(A-\alpha_{i}K_{i,k}H_{i,k}\right)}^{T}P_{i,k+1}^{-1}L_{i,k}\sum_{j\in N_{i}}\left(e_{j,k}^{a}-e_{i,k}\right)\right\}\\ \; & +\sum_{i=1}^{M}\alpha_{i}^{2}E\left\{v_{i,k}^{T}K_{i,k}^{T}P_{i,k+1}^{-1}K_{i,k}v_{i,k}\right\}+\sum_{i=1}^{M}E\left\{w_{k}^{T}P_{i,k+1}^{-1}w_{k}\right\} \end{array}$$

In order to simplify the terms on the right side of Equation (25), we first utilize Assumption 2 to scale the filter gain matrix in (19) to

(26) $$\begin{array}{c} ∥K_{i,k}∥=∥\alpha_{i}^{-1}AP_{i,k}H_{i,k}^{T}{\left(H_{i,k}P_{i,k}H_{i,k}^{T}+R_{i,k}\right)}^{-1}∥\leq \bar{a}{\bar{p}}_{i}{\bar{h}}_{i}/\alpha_{i}{\underline{h}}_{i}^{2}{\underline{p}}_{i} \end{array}$$

and the same approximation can be used by

(27) $$\begin{array}{cc} ∥A-\alpha_{i}K_{i,k}H_{i,k}∥= & \;∥A-AP_{i,k}H_{i,k}^{T}{\left(H_{i,k}P_{i,k}H_{i,k}^{T}+R_{i,k}\right)}^{-1}H_{i,k}∥\\ = & \;∥AP_{i,k}\left(\frac{1}{P_{i,k}}-\frac{H_{i,k}H_{i,k}^{T}}{H_{i,k}P_{i,k}H_{i,k}^{T}+R_{i,k}}\right)∥\\ = & \;∥\frac{AR_{i,k}}{H_{i,k}P_{i,k}H_{i,k}^{T}+R_{i,k}}∥\leq \bar{a}{\bar{r}}_{i}/\left({\underline{h}}_{i}^{2}{\underline{p}}_{i}+{\underline{r}}_{i}\right) \end{array}$$

Then, it follows from Equation (19) and Inequality (27) that

(28) $$\begin{array}{cc} P_{i,k+1}\geq & \left(A-\alpha_{i}K_{i,k}H_{i,k}\right)P_{i,k}{\left(A-\alpha_{i}K_{i,k}H_{i,k}\right)}^{T}+Q_{k}\\ \geq & \left(A-\alpha_{i}K_{i,k}H_{i,k}\right)\left[P_{i,k}+\frac{\underline{q}}{\frac{{\bar{p}}_{i}{\bar{a}}^{2}{\bar{r}}_{i}^{2}}{{\left({\underline{h}}_{i}^{2}{\underline{p}}_{i}+{\underline{r}}_{i}\right)}^{2}}}P_{i,k}\right]{\left(A-\alpha_{i}K_{i,k}H_{i,k}\right)}^{T} \end{array}$$

Further, Inequality (28) is rearranged into

(29) $$\begin{array}{cc} {\left[1+\frac{\underline{q}{\left({\underline{h}}_{i}^{2}{\underline{p}}_{i}+{\underline{r}}_{i}\right)}^{2}}{{\bar{p}}_{i}{\bar{a}}^{2}{\bar{r}}_{i}^{2}}\right]}^{-1}P_{i,k}^{-1}\geq & \left(A-\alpha_{i}K_{i,k}H_{i,k}\right)P_{i,k+1}^{-1}{\left(A-\alpha_{i}K_{i,k}H_{i,k}\right)}^{T} \end{array}$$

Thus, the first term of the Lyapunov function expansion in Equation (25) can be scaled as

(30) $$\begin{array}{cc} \; & \sum_{i=1}^{M}E\left\{e_{i,k}^{T}{\left(A-\alpha_{i}K_{i,k}H_{i,k}\right)}^{T}P_{i,k+1}^{-1}\left(A-\alpha_{i}K_{i,k}H_{i,k}\right)e_{i,k}\right\}\\ \; & \;\;\leq {\left[1+\frac{\underline{q}{\left({\underline{h}}_{i}^{2}{\underline{p}}_{i}+{\underline{r}}_{i}\right)}^{2}}{{\bar{p}}_{i}{\bar{a}}^{2}{\bar{r}}_{i}^{2}}\right]}^{-1}E\left\{V_{k}\left(e_{k}\right)\right\} \end{array}$$

By defining the consensus gain matrix as $L_{i,k}=\rho P_{i,k+1}{\left[{\left(A-\alpha_{i}K_{i,k}H_{i,k}\right)}^{T}\right]}^{-1}$, the third and fourth terms of the Lyapunov function expansion in Equation (25) are further arranged into

(31) $$\begin{array}{cc} \; & \sum_{i=1}^{M}E\left\{\sum_{j\in N_{i}}{\left(e_{j,k}^{a}-e_{i,k}\right)}^{T}L_{i,k}^{T}P_{i,k+1}^{-1}\left(A-\alpha_{i}K_{i,k}H_{i,k}\right)e_{i,k}\right\}\\ \; & \;+\sum_{i=1}^{M}E\left\{e_{i,k}^{T}{\left(A-\alpha_{i}K_{i,k}H_{i,k}\right)}^{T}P_{i,k+1}^{-1}L_{i,k}\sum_{j\in N_{i}}\left(e_{j,k}^{a}-e_{i,k}\right)\right\}\\ \; & =2\sum_{i=1}^{M}E\left\{e_{i,k}^{T}{\left(A-\alpha_{i}K_{i,k}H_{i,k}\right)}^{T}P_{i,k+1}^{-1}L_{i,k}\sum_{j\in N_{i}}\left(e_{j,k}^{a}-e_{i,k}\right)\right\}\\ \; & =2\rho \sum_{i=1}^{M}E\left\{e_{i,k}^{T}\sum_{j\in N_{i}}\left(e_{j,k}^{a}-e_{i,k}\right)\right\}\\ \; & \leq \rho {\bar{p}}_{i}E\left\{V_{k}\left(e_{k}\right)\right\}+\rho \sum_{i=1}^{M}E\left\{{\left(e_{j,k}^{a}-e_{i,k}\right)}^{T}\left(e_{j,k}^{a}-e_{i,k}\right)\right\}\\ \; & \leq \rho {\bar{p}}_{i}E\left\{V_{k}\left(e_{k}\right)\right\}+\rho \sigma \end{array}$$

where $\sigma =\sum_{i=1}^{M}\sigma_{i}$. $\sigma_{i}$ is determined by the following inequality:

$$\begin{array}{c} \sum_{j\in N_{i}}{\left({\hat{X}}_{j,k}^{a}-{\hat{X}}_{i,k}\right)}^{T}\left({\hat{X}}_{j,k}^{a}-{\hat{X}}_{i,k}\right)\leq \sigma_{i} \end{array}$$

with $\sigma_{i}>0,i=1,2,\cdots ,M$.

In a similar way, the second term of the Lyapunov function expansion in Equation (25) yields

(32) $$\begin{array}{cc} \; & \sum_{i=1}^{M}E\left\{\sum_{j\in N_{i}}{\left(e_{j,k}^{a}-e_{i,k}\right)}^{T}L_{i,k}^{T}P_{i,k+1}^{-1}L_{i,k}\left(e_{j,k}^{a}-e_{i,k}\right)\right\}\\ \; & =\sum_{i=1}^{M}E\left\{L_{i,k}^{T}P_{i,k+1}^{-1}L_{i,k}\right\}E\left\{{\left(e_{j,k}^{a}-e_{i,k}\right)}^{T}\left(e_{j,k}^{a}-e_{i,k}\right)\right\}\\ \; & \leq \frac{\rho^{2}\sigma {\left({\underline{h}}_{i}^{2}{\underline{p}}_{i}+{\underline{r}}_{i}\right)}^{2}}{{\bar{a}}^{2}{\bar{r}}_{i}^{2}} \end{array}$$

Next, the remaining noise terms in the Lyapunov function expansion is addressed:

(33) $$\begin{array}{cc} \sum_{i=1}^{M}\alpha_{i}^{2}E\left\{v_{i,k}^{T}K_{i,k}^{T}P_{i,k+1}^{-1}K_{i,k}v_{i,k}\right\}\leq & \sum_{i=1}^{M}\frac{{\bar{a}}^{2}{\bar{p}}_{i}^{2}{\bar{h}}_{i}^{2}{\bar{r}}_{i}}{{\underline{h}}_{i}^{4}{\underline{p}}_{i}^{3}} \end{array}$$

(34) $$\begin{array}{cc} \sum_{i=1}^{M}E\left\{w_{k}^{T}P_{i,k+1}^{-1}w_{k}\right\}\leq & \sum_{i=1}^{M}\frac{\bar{q}}{{\underline{p}}_{i}} \end{array}$$

Finally, through the combination of Inequalities (30)–(34), we can draw the conclusion as

(35) $$\begin{array}{cc} E\{V_{k+1}\left(e_{k+1}\right)\}\leq & \left\{{\left[1+\frac{\underline{q}{\left({\underline{h}}_{i}^{2}{\underline{p}}_{i}+{\underline{r}}_{i}\right)}^{2}}{{\bar{p}}_{i}{\bar{a}}^{2}{\bar{r}}_{i}^{2}}\right]}^{-1}+\rho {\bar{p}}_{i}\right\}E\left\{V_{k}\left(e_{k}\right)\right\}\\ \; & +[\rho \sigma +\frac{\rho^{2}\sigma {\left({\underline{h}}_{i}^{2}{\underline{p}}_{i}+{\underline{r}}_{i}\right)}^{2}}{{\bar{a}}^{2}{\bar{r}}_{i}^{2}}+\sum_{i=1}^{M}\frac{{\bar{a}}^{2}{\bar{p}}_{i}^{2}{\bar{h}}_{i}^{2}{\bar{r}}_{i}}{{\underline{h}}_{i}^{4}{\underline{p}}_{i}^{3}}+\sum_{i=1}^{M}\frac{\bar{q}}{{\underline{p}}_{i}}] \end{array}$$

By comparing Inequalities (21) and (35), it can be found that the second condition in Lemma 1 satisfies when

(36) $$\begin{array}{cc} \kappa = & 1-{\left[1+\frac{\underline{q}{\left({\underline{h}}_{i}^{2}{\underline{p}}_{i}+{\underline{r}}_{i}\right)}^{2}}{{\bar{p}}_{i}{\bar{a}}^{2}{\bar{r}}_{i}^{2}}\right]}^{-1}-\rho {\bar{p}}_{i} \end{array}$$

(37) $$\begin{array}{cc} \upsilon = & \rho \sigma +\frac{\rho^{2}\sigma {\left({\underline{h}}_{i}^{2}{\underline{p}}_{i}+{\underline{r}}_{i}\right)}^{2}}{{\bar{a}}^{2}{\bar{r}}_{i}^{2}}+\sum_{i=1}^{M}\frac{{\bar{a}}^{2}{\bar{p}}_{i}^{2}{\bar{h}}_{i}^{2}{\bar{r}}_{i}}{{\underline{h}}_{i}^{4}{\underline{p}}_{i}^{3}}+\sum_{i=1}^{M}\frac{\bar{q}}{{\underline{p}}_{i}} \end{array}$$

where $0<{\left[1+\frac{\underline{q}{\left({\underline{h}}_{i}^{2}{\underline{p}}_{i}+{\underline{r}}_{i}\right)}^{2}}{{\bar{p}}_{i}{\bar{a}}^{2}{\bar{r}}_{i}^{2}}\right]}^{-1}+\rho {\bar{p}}_{i}\leq 1$.

In conclusion, the results confirmed that the estimation error $e_{i,k}$ is bounded with probability one and exponentially bounded in mean square, which completes this proof. □

## 6. Simulation Results

In this section, the IEEE 14-bus power system is introduced as a case study to verify the proposed algorithms. Taking into account the model of distributed power systems akin to [36], the related parameters are listed below:

$$\begin{array}{cc} \; & A={\mathrm{diag}}_{28}\left\{0.98\right\},\;B={\mathrm{diag}}_{28}\left\{0.02\right\},\\ \; & Q\left(k\right)={\mathrm{diag}}_{28}\left\{0.01\right\} \end{array}$$

where $X_{0}={\left[X_{0}^{r},X_{0}^{i}\right]}^{T}$ with $X_{0}^{r}=1_{14}^{T}$ p.u and $X_{0}^{i}=0_{14}^{T}$ p.u. The value of *u* in (1) is given by [36,39].

**Figure 1:**
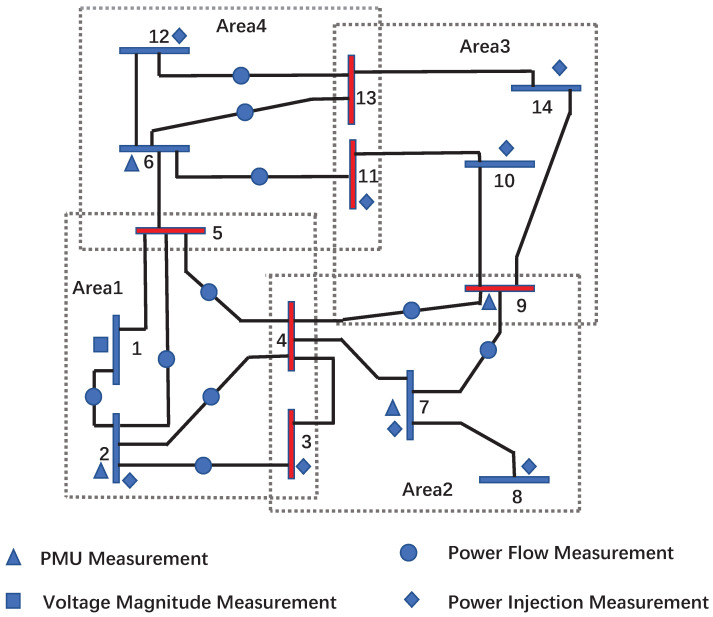
IEEE 14-bus system partitioned in 4 areas.

In addition, the measurement configuration of the experimental example comes from [45], which is shown in Figure 1. The measurement system includes both conventional RTUs and advanced PMUs, where RTU measurements are categorized into three different parts as follows:

1. The voltage magnitude data measured from bus 1;
2. Power injection data measured from buses 2, 3, 7, 8, 10, 11, 12, and 14;
3. Power flow data measured between bused 1 and 2, 2 and 3, 4 and 2, 4 and 7, 4 and 9, 5 and 2, 5 and 4, 5 and 6, 6 and 13, 7 and 9, 11 and 6, as well as 12 and 13.

Furthermore, PMUs are placed on buses 2,6,7, and 9, allowing measurements of their voltage and current data.

In order to obtain the distributed measurement model described by Equation (12), the IEEE-14 bus power system shown in Figure 1 is divided into four interconnected subsystems.

**Figure 2:**
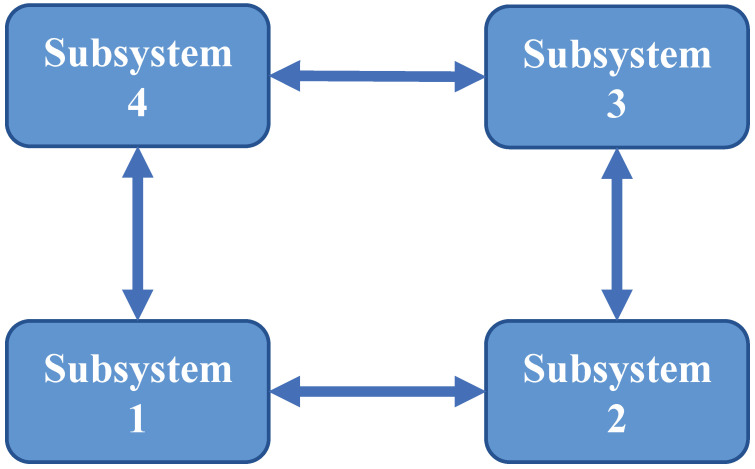
The communication topology of the interconnected subsystems.

The communication topology of the interconnected subsystems is simplified as shown in Figure 2, where the FDI attack is considered to occur between subsystems 1 and 2, and the DoS attack is considered to occur in all subsystems. In addition, the measurement noise covariances of all subsystems are set to $R_{i,k}=0.01I$.

**Figure 3:**
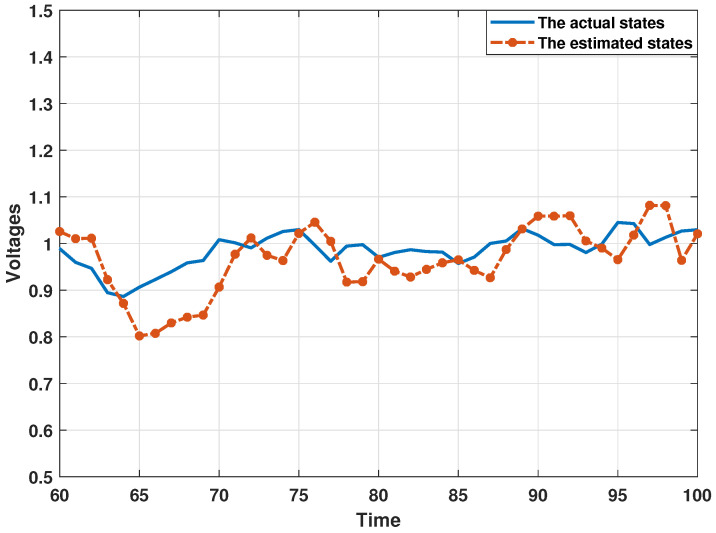
The estimated values from the presented algorithm under collaborative attacks for the real parts of states at bus 1.

**Figure 4:**
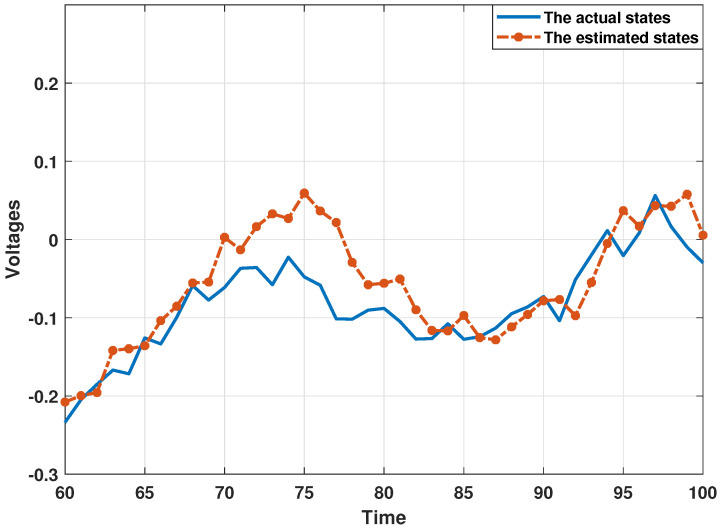
The estimated values from the presented algorithm under collaborative attacks for the imaginary parts of states at bus 1.

**Figure 5:**
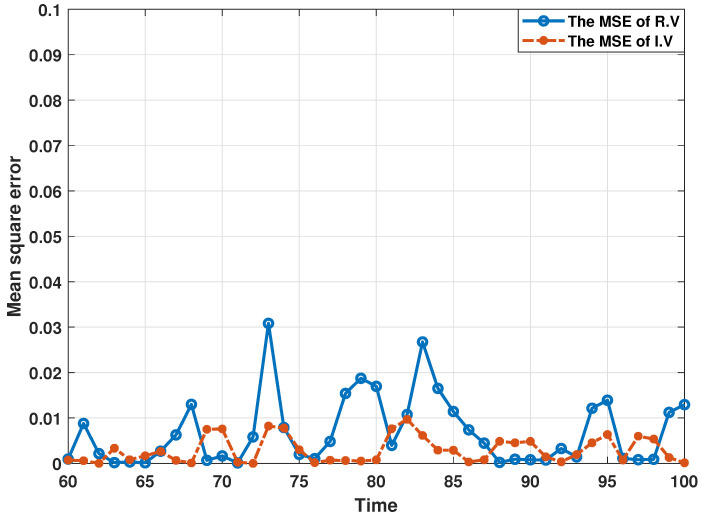
MSEs of the estimated real and imaginary parts of states at bus 1 under collaborative attacks.

In order to demonstrate the estimation performance of the proposed distributed consensus estimation algorithm, the following experiments are all performed under the influence of collaborative attacks, where the probability that the transmitted innovation is not subject to the DoS attack is set as $\alpha_{i}=0.9$, and the covariance matrix of the FDI attack variable is set as $B_{ij,k}=0.5I$. Then, this section presents the estimation results in Figure 3 and Figure 4 by using bus 1 as a representative bus. It can be found that the estimates of the real and imaginary parts of the state of bus 1 can track the actual state well, which shows that the proposed distributed estimation algorithm can effectively perform the estimation process under the influence of collaborative attacks. In addition, in order to analyze the estimation performance more thoroughly and eliminate the influence of other factors, this section uses the Monte Carlo simulation method in [36,39] to present the estimation results. In the Monte Carlo simulation method, the mean square error (MSE) is often used to describe the simulation results, which is defined in this paper as ${\mathrm{MSE}}_{i}\left(k\right)=\frac{1}{N}\sum_{j}^{N}\left(X_{k}-{\hat{X}}_{i,k}\right)$ with N=100. Here, the MSEs of the estimated real and imaginary parts of states from estimator 1 are shown in Figure 5. It can be seen that all MSEs are kept in a small range, which also illustrates the effectiveness of the proposed distributed estimation algorithm.

**Figure 6:**
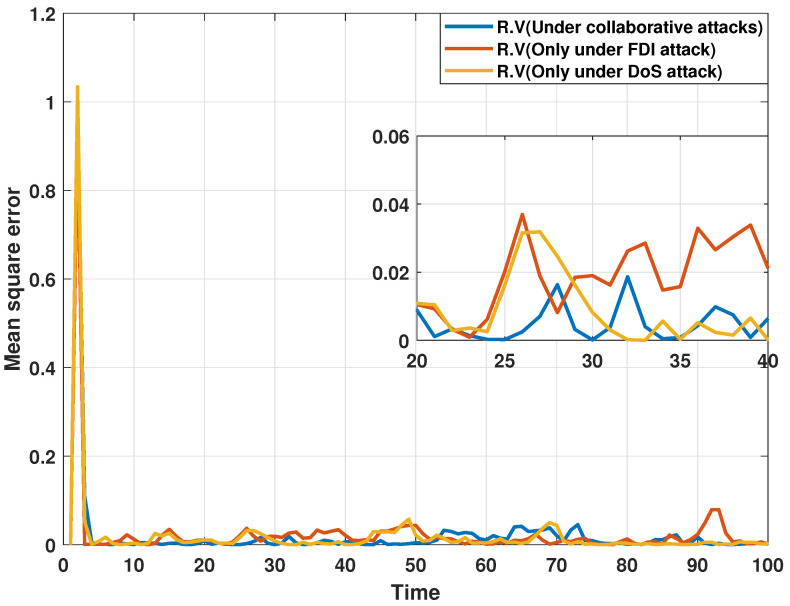
MSEs of the estimated real parts of states at bus 1 under different attack cases.

**Figure 7:**
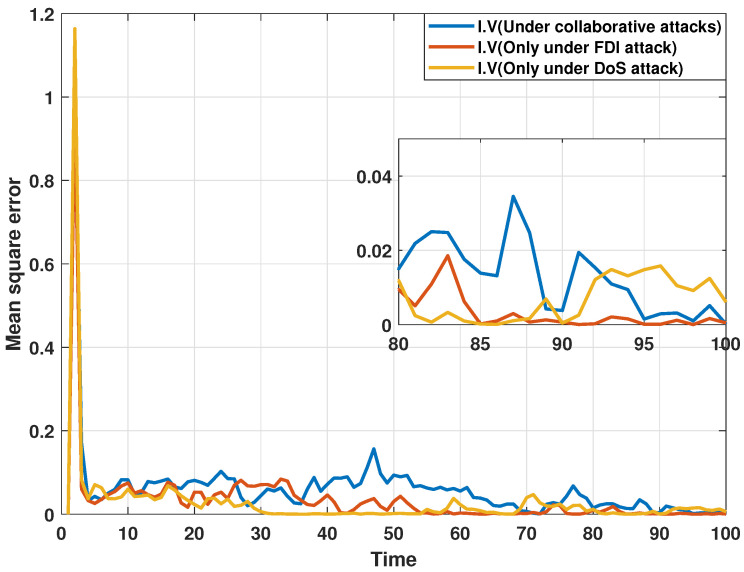
MSEs of the estimated imaginary parts of states at bus 1 under different attack cases.

On the other hand, the estimation performance of the proposed distributed consensus estimation algorithm is compared under three attack cases. The first case, from [33], proposed a distributed estimator that is only subject to FDI attacks. The second case, from [34], focuses only on DoS attacks. The last case is from this paper. In order to show the results of the comparison, MSEs of the estimated real and imaginary parts of states at bus 1 under different attack cases are plotted in Figure 6 and Figure 7, respectively. It can be discovered that MSEs does not change significantly under the three different attack scenarios, which shows that the proposed distributed estimation algorithm can maintain the estimation accuracy under a single type of attack while resisting multiple types of collaborative attacks, indicating that the proposed algorithm has high robustness.

**Figure 8:**
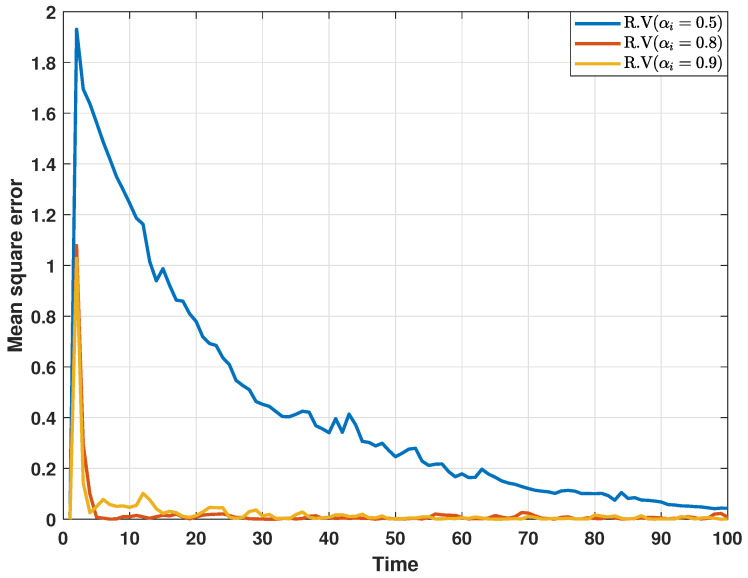
MSEs of the estimated real parts of states at bus 1 under different DoS attack probabilities.

**Figure 9:**
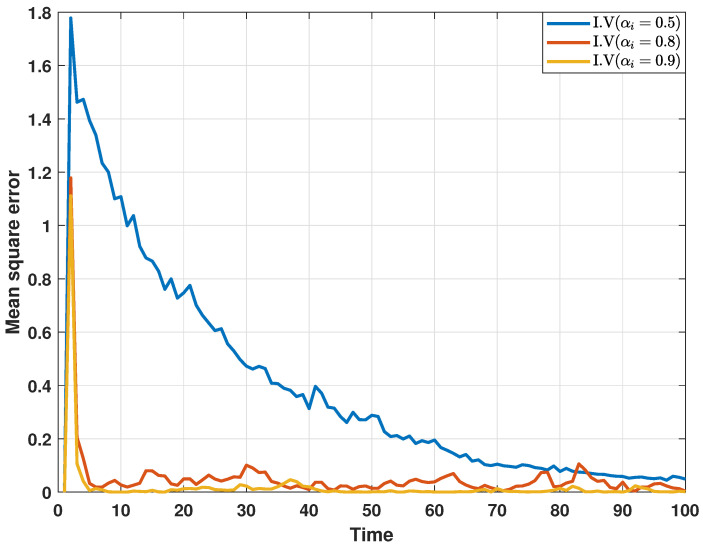
MSEs of the estimated imaginary parts of states at bus 1 different DoS attack probabilities.

In addition, in order to further analyze the impact of collaborative attacks on the performance of the proposed distributed consensus estimation algorithm, a comparative experiment is performed under different DoS attack probabilities, and the estimation results are depicted in Figure 8 and Figure 9. Here, $\alpha_{i}$ represents the probability that the transmitted innovation is not attacked.In other words, as the attack probability increases, the estimation performance becomes worse, which confirms that collaborative attacks will cause performance loss to the estimator.

**Figure 10:**
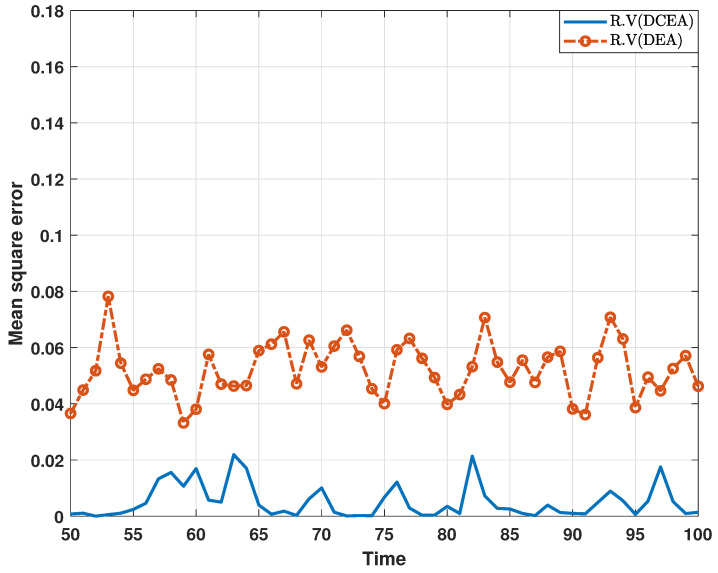
MSEs of the estimated real parts of states at bus 1 under DCEA and DEA.

**Figure 11:**
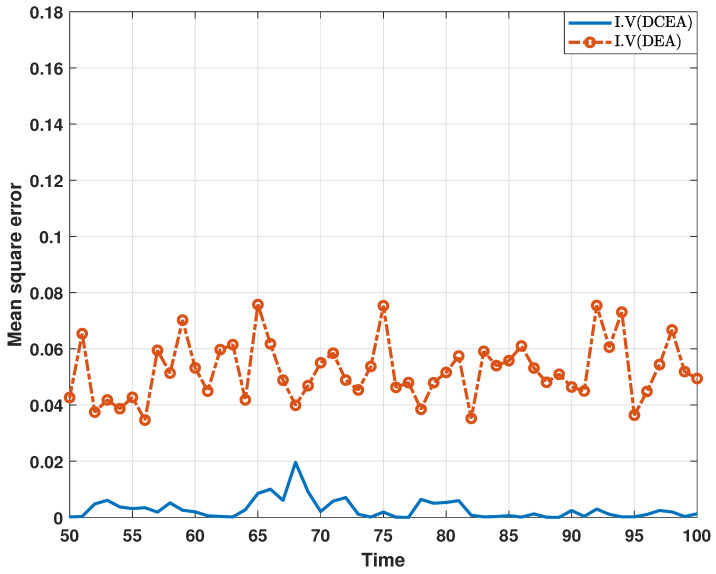
MSEs of the estimated imaginary parts of states at bus 1 under DCEA and DEA.

In the following, a comparative experiment on the estimation performance of the proposed algorithm and existing algorithm is performed. In Figure 10 and Figure 11, the comparison results with respect to the MSEs of the estimated real parts of states are depicted, where the distributed consensus estimation algorithm proposed in this paper is referred to as “DCEA”, and is implemented as shown in (19), and the other algorithm is referred to as “DEA”, and is implemented for each node in an isolated way [24]. According to the results, it can be found that the proposed distributed consensus estimation algorithm performs better than the existing algorithm. In particular, the consensus term helps to improve estimation performance when compared to the DEA in [24].

## 7. Conclusions

This paper has presented a consensus-based distributed dynamic state estimation algorithm for power systems subject to collaborative attacks. According to the hybrid RTU and PMU measurements, as well as the neighboring estimation, DoS and FDI attack models have been constructed using random variables that satisfy the Gaussian distribution and the Bernoulli distribution, respectively. By incorporating a consensus strategy into the distributed Kalman filter, a distributed consensus estimator has been devised to improve the estimation performance of power systems under collaborative attacks. A suboptimal distributed estimator, as a simplified version of the optimal one, has been proposed to reduce the computational cost. A sufficient condition and a consensus gain matrix have been properly designed to ensure the stability of the proposed estimator. Future works will focus on extending linear multi-sensor systems to nonlinear systems. In addition, extending linear power systems to nonlinear systems will be the main focus of future research. In addition, the redundancy of system observability under sparse sensor attacks in [46] is also a focus of future attention.

## Data Availability

The original contributions presented in the study are included in the article, further inquiries can be directed to the corresponding author.

## Abbreviations

The following abbreviations are used in this manuscript:

RTU	remote terminal unit
PMU	phasor measurement unit
FDI	false data injection
DoS	denial of service
MSE	mean square error
